# Mindful Yoga for women with metastatic breast cancer: design of a randomized controlled trial

**DOI:** 10.1186/s12906-017-1672-9

**Published:** 2017-03-13

**Authors:** James W. Carson, Kimberly M. Carson, Maren K. Olsen, Linda Sanders, Laura S. Porter

**Affiliations:** 1 Department of Anesthesiology & Perioperative Medicine, Oregon Health & Science University, 3181 SW Sam Jackson Park Rd., Portland, OR 97239 USA; 2 Center for Health Services Research in Primary Care, Durham VA Medical Center, Durham, NC 27705 USA; 3 Department of Medicine, Duke University Medical Center, Box 2628, Durham, NC 27710 USA; 4 Department of Psychiatry and Behavioral Sciences, Duke University Medical Center, Box 90399, Durham, NC 27708 USA

**Keywords:** Breast cancer, Cancer pain, Yoga, Mindfulness, Research design

## Abstract

**Background:**

Women with metastatic breast cancer (MBC) have average life expectancies of about 2 years, and report high levels of disease-related symptoms including pain, fatigue, sleep disturbance, psychological distress, and functional impairment. There is growing recognition of the limitations of medical approaches to managing such symptoms. Yoga is a mind-body discipline that has demonstrated a positive impact on psychological and functional health in early stage breast cancer patients and survivors, but has not been rigorously studied in advanced cancer samples.

**Methods:**

This randomized controlled trial examines the feasibility and initial efficacy of a Mindful Yoga program, compared with a social support condition that controls for attention, on measures of disease-related symptoms such as pain and fatigue. The study will be completed by December 2017. Sixty-five women with MBC age ≥ 18 are being identified and randomized with a 2:1 allocation to Mindful Yoga or a support group control intervention. The 120-min intervention sessions take place weekly for 8 weeks. The study is conducted at an urban tertiary care academic medical center located in Durham, North Carolina. The primary feasibility outcome is attendance at intervention sessions. Efficacy outcomes include pain, fatigue, sleep quality, psychological distress, mindfulness and functional capacity at post-intervention, 3-month follow-up, and 6-month follow-up.

**Discussion:**

In this article, we present the challenges of designing a randomized controlled trial with long-term follow-up among women with MBC. These challenges include ensuring adequate recruitment including of minorities, limiting and controlling for selection bias, tailoring of the yoga intervention to address special needs, and maximizing adherence and retention. This project will provide important information regarding yoga as an intervention for women with advanced cancer, including preliminary data on the psychological and functional effects of yoga for MBC patients. This investigation will also establish rigorous methods for future research into yoga as an intervention for this population.

**Trial registration:**

ClinicalTrials.gov identifer: NCT01927081, registered August 16, 2013

## Background

Although metastatic breast cancer (MBC) is a terminal illness, the development of more effective and better tolerated therapies has led to improved prognosis, with average life expectancies of 2 years or more. Nonetheless, women with MBC continue to report poor quality of life and high levels of pain and other disease-related symptoms including fatigue, sleep disturbance, psychological distress, and functional impairment [1]. Coping with these symptoms in the context of a life-limiting disease is very challenging [2, 3]. There is growing recognition of the limitations of medical approaches to managing cancer pain and related symptoms, and heightened interest in the role that self-management interventions can play in cancer symptom control [4]. Considering the challenging course of MBC along with the high prevalence of pain and associated symptoms and impairments, it is critical to develop effective interventions to enhance the quality of the remaining years of these women’s lives.

Recently there has been a growing interest in incorporating mind/body practices such as yoga and tai chi to help cancer patients cope with disease-related symptoms [5, 6]. The Office of Cancer Complementary and Alternative Medicine has prioritized studies of yoga and other mind/body therapies, as has the National Center for Complementary and Integrative Health [7, 8]. Yoga has been practiced in India for its proposed physical and mental benefits for thousands of years [9]. Yoga (which means ‘union’ in Sanskrit) aims at uniting mind and body through a variety of strategies such as practicing poses that promote strength, balance and flexibility [10–12]; breathing techniques that have soothing and energizing effects [13, 14]; and meditation to develop mental calm and emotional clarity [15].

Over the last several decades yoga interventions have been demonstrated to positively impact a variety of medical conditions, including hypertension [16, 17], asthma [18], multiple sclerosis [19], tuberculosis [20], back pain [21, 22], fibromyalgia [23, 24], and neck pain [25]. Yoga has also shown promise for improving cancer-related symptoms such as fatigue and stress [26]. However prior studies in cancer patients have been largely limited to early stage breast cancer patients and survivors. Thus far, five systematic reviews of randomized controlled trials (RCTs) of yoga for cancer have been published [27–31]. Three reviews included meta-analyses [27, 28, 30], and one of the remaining two focused exclusively on fatigue outcomes [31]. The number of RCTs included across the reviews ranged from 10 to 18, with sample sizes ranging from 18 to 164. Although the research quality across these studies varied greatly, on average the quality was relatively good (e.g., median quality = 67%, range 22–89% [27]). Most of the reviewed yoga interventions primarily emphasized physical poses, supplemented by some combination of breathing techniques, and relaxation or meditation practices. Approximately 50% of RCTs compared yoga with wait-list control conditions, and most of the remaining studies employed supportive care control conditions [29].

The consensus conclusion across the reviews was that there is moderate to good evidence that, at least in the short-term, yoga has a positive impact on psychological health in early stage breast cancer patients and survivors. Effect sizes for psychosocial outcomes (e.g., emotional distress, anxiety, depression, global quality of life) were moderate to large, whereas for functional well-being effect sizes were small. Fatigue improved in approximately 50% of studies where assessed. Only one RCT—which was conducted by the authors of this paper—assessed pain, which improved in that study [32]. None of the studies reported serious adverse events. Each of the available systematic reviews concluded that larger, more rigorous trials are needed and that these trials should include methodological improvements such as better control conditions, more physiological measures, longer term follow-up, and a wider range of cancer populations, including those with advanced cancer. A notable gap in the yoga for breast cancer literature is the absence of RCTs among individuals with advanced cancer. Recently, we conducted a pilot study to test a novel 8-week Mindful Yoga intervention designed to address the cancer-related pain, fatigue and emotional distress experienced by women with MBC. This intervention (previously published as ‘Yoga of Awareness’) showed promising results in terms of effects on these symptoms, and feasibility [33]. However, the MBC pilot study had major limitations including the lack of a control group, very small sample size (*N* = 13), and failure to address mediators that might explain the treatment effects.

In this paper, we present the design and detailed protocol of the first-known RCT of a yoga intervention for patients with advanced cancer. This single-blinded, randomized, attention-controlled, clinical trial for women with MBC has been funded by the National Center for Complementary and Integrative Health. Along with design considerations, we discuss the overall challenges of this trial as regards recruitment strategies, tailoring of the yoga intervention, and maximizing adherence and retention. We describe methods for addressing the theoretical and logistical issues encountered in conducting such a trial. Upon completion of the study, the results will be reported in accordance with the Consolidation of Standards for Reporting Trials (CONSORT) guidelines [34].

## Methods/Design

### Study Design

This is a randomized, controlled, single-blind, clinical trial of yoga in the treatment of cancer-related pain and associated symptoms in 65 women with metastatic breast cancer. Given the absence of prior yoga RCTs for patients with advanced cancer, our aims encompass the feasibility and acceptability of the Mindful Yoga intervention in this population, as well as comparing the efficacy of this intervention with a social support comparison condition. Feasibility will be indicated by our ability to meet accrual goals, and by at least 70% of patients attending ≥ 4 of 8 sessions and providing post-test assessments. Acceptability will be indicated by ≥ 80% of participants reporting satisfaction with the yoga intervention on a standardized measure of treatment effectiveness/satisfaction. Regarding efficacy, we hypothesize that the yoga intervention will lead to reductions in pain, fatigue, sleep disturbance, and psychological distress and increases in functional capacity compared to a social support intervention.

The social support comparison was chosen for several reasons. First, we sought to address literature reviewers’ criticism of the frequent employment of treatment-as-usual and wait-list control conditions in yoga for cancer RCTs [27–31]. Such comparisons fail to control for any factor other than the general passage of time. Second, based on our prior research and reviewer’s recommendations, we determined that the most important factors to control for were attention and time within a professionally-guided group intervention context. We ultimately decided on a social support comparison condition because previous studies indicate this type of intervention is viewed as highly credible by patients, and attendance at social support groups is comparable to that of other intervention groups [35–37]. Along with controlling for attention and time, this condition controls for nonspecific treatment effects such as general social support. The “single-blind” study design was chosen because it was not feasible to conceal assignment to the yoga intervention from participants and the intervention instructors. However, all study investigators and study staff involved in data collection will be masked to treatment condition, and statisticians will remain masked until the database is deemed final and ready for statistical analysis. As this is a low-risk behavioral trial, we do not foresee any circumstance under which blinded staff members will need to be unblinded during the trial.

Outcome assessments include intervention attendance rates, self-report measures of intervention satisfaction, pain, fatigue, sleep quality, psychological distress, mindfulness, a 6 min walk test measuring functional capacity, and treatment expectations. Assessments will be completed at baseline, post-treatment, and 3-month and 6-month follow-up. In addition, during the intervention period, participants will complete brief daily diaries on alternate weeks (4 weeks total) assessing symptoms (pain, fatigue, positive mood, negative mood, pain catastrophizing, pain acceptance) and yoga practice. An innovative feature of this study is that we will utilize these daily measures to conduct a rigorous test of two key psychological mediators that may explain the mechanisms of treatment effects. Pain theory and research point to two variables—pain catastrophizing (the tendency to focus on and exaggerate the threat value of painful stimuli and negatively evaluate one’s own ability to deal with pain) and pain acceptance (the willingness to experience pain and yet remain engaged in meaningful life activities despite pain)—that are likely to be particularly important in explaining the effects of the yoga intervention on pain and pain-related outcomes [38, 39]. Understanding the underlying mechanisms of the yoga intervention’s potential effects could have far-reaching implications for the design and evaluation of future yoga interventions for conditions entailing persistent pain.

**Fig. 1:**
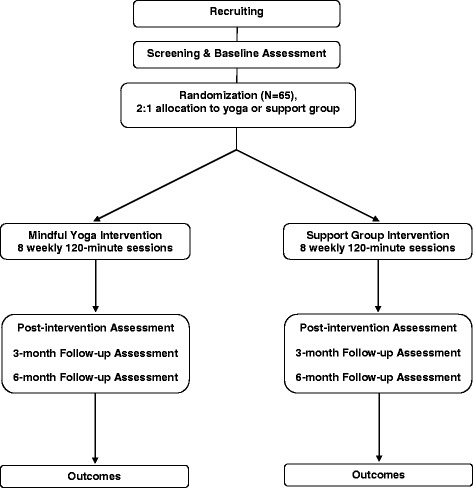
Study flow chart

The setting of the study is an urban tertiary care academic medical center located in Durham, North Carolina (Duke Cancer Institute). This study has been reviewed and approved by the Duke Medical Center Institutional Review Board (IRB). The study will adhere to the CONSORT guidelines for clinical trials [34]. The study flow is presented in Fig. 1.

### Study population

Participants in this study are women with age ≥ 18 who are receiving treatment for metastatic breast cancer (Stage IV breast cancer or recurrent metastatic breast cancer). Participants must have a life expectancy ≥ 9 months as estimated by their treating oncologist; speak and read English; and be able to understand study procedures and comply with them for the entire length of the study period. Individuals will be excluded if they have: (a) cognitive impairment as assessed by the 6-item Mini-Mental Status Exam; (b) Eastern Cooperative Oncology Group (ECOG) rating of ≥ 3 or Karnofsky Performance Status (KPS) < 60 as rated by the oncology provider; (c) too sick to participate, as determined by the treating oncologist; (d) received treatment for serious psychiatric illness (e.g., schizophrenia, severe depression) in the past 6 months; (e) current engagement in yoga practice ≥ 1 day per week; (f) resting systolic blood pressure > 180 mm and/or diastolic blood pressure > 100 mm, or resting heart rate > 100 beats per minute at the baseline assessment; (g) unable or unwilling to give written informed consent.

During the trial, all participants will continue to receive the usual care provided by their health care providers. Medications and treatments received during the study are documented and examined later in data analyses, but not restricted.

### Recruitment strategies

The percentage of MBC patients that accept entry when approached about behavioral intervention studies varies widely in the literature, from 27% [40] to 84% [41]. Our recruitment strategies focus on enrolling interested, completely informed participants, and we have projected a modest 25% acceptance rate for the present study. To address the significant gaps in research participation among minorities that limit the generalizability of findings, especially among African Americans (38% of the population in Durham County, North Carolina [42]), we are employing a variety of strategies to optimize minority participant involvement in the investigation.

Initial contact with potential participants is initiated by each patient’s health care provider, either verbally or by an IRB-approved introductory letter and pamphlet. Given the rapidly expanding interest in yoga among breast cancer patients [43], the introductory materials describe the yoga intervention as a “self-management discussion group + gentle exercise”, and the social support intervention as a “self-management discussion group”, with the aim of minimizing selection bias related to patients’ intervention preferences. This introduction includes graphics that are inclusive of various minority groups. Consistent with recommendations on the recruitment of African-American participants for clinical and prevention trials, particularly in oncology, we provide African-American (and all) potential participants with an opportunity to review the information about the study without any direct request to participate. Individuals who do not refuse further contact are called by a clinical research coordinator who has received training in how to address the social and cultural barriers to engaging African American cancer survivors in behavioral treatment studies. The study coordinator provides a brief scripted explanation of the study, answers any questions, and verifies eligibility. If the patient is eligible and chooses to participate, arrangements are made to obtain written consent and to administer the baseline assessment. As an added incentive, each participant is paid $190 for full participation in the study to offset costs participants might incur by participating (e.g., babysitters during sessions) and as an honorarium for their time.

### Adherence and retention strategies

Adherence and retention are crucial determinants of the quality and interpretability of clinical trials. Obstacles to treatment attendance when intervening with patients with advanced disease include a variety of illness-related issues, such as unpredictable worsening of symptoms, limited energy, and alternate patient priorities given shortened life expectancies. Strategies we have implemented to boost attendance are among the best practices used in many high quality behavioral intervention trials. These strategies include motivational interviewing during the recruitment phase, weekly reminder emails during the intervention phase, prompt calls from interventionists to participants who missed a class, and make up sessions. Adequate adherence in this trial has been defined as 70% of the participants attending full sessions (120 min each) for at least 4 of the 8 treatment sessions. This is a revision from the initial definition of adequate adherence, which specified 70% attendance of at least 7 intervention sessions. We believe that the revision represents a more reasonable expectation given the severity of illness in this patient population. In reviewing data from three previous studies of the 8-session Mindful Yoga intervention, it was apparent that the goal of 70% of participants attending at least 7 sessions was likely overly ambitious [32, 33, 44]. This was achieved in only one of the three previous studies, with a population of much healthier women who were post-treatment for early stage breast cancer [32].

Attrition is a recognized challenge in studying the MBC population, given the progressive nature of the disease. To minimize missing data, we developed a retention plan to ensure minimal dropout due to reasons other than death or severe disease progression. This plan has featured minimization of participant burden, provision of participant support through the intervention protocols, solicitation of information about participant difficulties and prompt response to concerns raised, and consistent efforts to optimize the participants’ experience of study participation. Continuous quality monitoring of retention key performance indicators has allowed us to rapidly identify and respond to problems.

### Sample size

Our original sample size of 60 patients (40 intervention and 20 control) was based mainly on the primary aim of intervention feasibility and acceptability. While we have increased the targeted enrollment to approximately 65 patients, statistical power to detect significant intervention differences remains limited. Nonetheless, our sample size will be sufficient to conduct longitudinal analyses of the outcome variables to inform the design of a larger-scale, multisite trial.

### Randomization

Participants are randomized with a 2:1 allocation to: (a) Mindful Yoga, or (b) a support group control intervention. The study statisticians (M.O. & L.S.) generated the randomization scheme prior to the start of recruitment, which is kept in a study database which is not accessible by blinded study personnel. The study coordinator who executes the randomization schedule does not have access to the data and is not involved in the outcome assessments.

Due to the group nature of the interventions, enrollment has proceeded in cohorts. Randomization has been stratified within each cohort using variable block sizes of 3 and 6. The study coordinator informs each participant of their assigned treatment condition following the baseline assessment and ≤ 14 days prior to the start of the interventions.

We planned to conduct cohorts of 20–25 participants, so that each intervention group (two yoga groups and one social support group per cohort) would have between 5 and 10 group members. After the first two cohorts, we found that class size was an important factor in the yoga classes, which functioned best with at least five patients in attendance, but that class size did not appear to impact the functionality of the social support classes. Given that attendance at each class is usually less than 100%, it is necessary to assign at least seven patients to each yoga class to meet the expectation of usually having at least five patients in attendance. This necessitates enrolling at least 20 patients per cohort to fill two yoga classes and one social support class.

### Intervention

Both interventions consist of eight group sessions conducted weekly in 120-min sessions (Fig. 1).

#### Mindful Yoga Program

This intervention consists of eight 120-min weekly group sessions led by a certified yoga instructor who has extensive experience in teaching yoga techniques to medical patients. The Mindful Yoga Program is based in part on the Kripalu school of yoga, which is widely taught in the U.S. [45, 46]. Mindful Yoga can be distinguished from many current yoga styles in that it places strong emphasis on developing mindfulness via substantial meditation practice, breathing exercises, study of yoga philosophy, practitioner meetings, and informal application of mindful awareness in daily life [26]. Mindfulness entails strategies for developing greater moment-to-moment presence of mind, along with acceptance of and willingness to learn from stressful experiences, so as to begin to recognize clearly what choices contribute to more wellbeing versus suffering [39, 47, 48]. Accordingly, during posture exercises Mindful Yoga intensively highlights the inner dimensions of practice—such as nonreactive monitoring of sensory, mental and emotional fluctuations—along with proper alignment and breathing techniques. The yoga instructor follows a detailed intervention manual (Carson JW, Carson KM: Mindful Yoga Professional Training Manual, unpublished). Each session includes gentle postures (approx. 40 min), breathing techniques (10 min), meditation (25 min), presentations on the application of yogic principles to optimal coping (e.g., mind/body stress reactions; 20 min), and group discussions (e.g., experiences during meditation; 25 min). The yoga poses included in this protocol carry no more risk of injury than everyday activities such as climbing stairs. They have been specifically selected and tailored to meet the needs of women with MBC, and the instructor emphasizes gentle performance. Patients are supplied with yoga mats, eye pillows, and bolsters for doing poses. Participants are encouraged to practice yoga techniques at home, 15–30 min per day, 5–6 days per week, guided by professionally produced video recordings. Participants also receive brief session summary handouts each week, which include instructions for the informal application of yoga practice to daily life (e.g., in-the-moment acceptance of pain).

#### Social support comparison condition

This intervention consists of eight 120-min weekly group sessions led by a licensed clinical social worker experienced in leading groups and in working with patients with advanced cancer. The scheduling of the sessions is identical to that for the yoga intervention. The social support intervention is modeled after the protocol utilized by Breitbart and colleagues, and the therapist follows a detailed treatment manual [35]. The sessions focus on discussion of issues and themes that emerge for patients coping with MBC, including the following: coping with medical tests; communicating with healthcare providers; coping with family and friends; vocational issues; body image and physical functioning concerns; fears about future physical or psychological changes, recurrence, and mortality; and plans for the future. Utilizing a supportive approach, the therapist focuses on encouraging patients to share concerns related to the cancer diagnosis and treatment, to describe their experiences and emotions related to these experiences, to voice problems that they have in coping with cancer, and to offer support and advice to other group members.

### Measures

All participants complete assessments at baseline, post-treatment, and 3-month and 6-month follow-up (see Table 1). In addition, they complete daily diary measures during alternating weeks during the intervention period assessing symptoms and yoga practice (for those in the yoga condition).

**Table 1:** Sequence of trial measurements for primary and secondary outcomes

	Pre-Screening	Telephone Screening	Baseline	Treatment	Mid-Treatment	Treatment	Post-Treatment	3-Month Follow-up	6-Month Follow-up
VISIT, Time (weeks)	(-16-0)	(-13-0)	1 (-4–0)	2–4 (1–3)	5 (4)	6–9 (6–8)	10 (9)	11 (21)	12 (34)
Primary outcome measures									
Attendance at intervention sessions				X		X			
Client Satisfaction Questionnaire-8							X		
Secondary outcome measures									
Brief Pain Inventory-Short Form			X				X	X	X
Brief Fatigue Inventory			X				X	X	X
Pittsburg Sleep Quality Index			X				X	X	X
Hospital Anxiety & Depression Scale			X				X	X	X
Mindfulness questionnaire (FFMQ-SF)			X				X	X	X
Complementary/alternative medicine use			X				X	X	X
6-min Walk Test			X				X	X	X
Medical record review	X		X				X	X	X
ECOG or KPS	X								
Demographics			X						
Daily diary items				X		X			
Medication log			X		X		X	X	X
Health questionnaire			X						
Treatment expectations			X		X				
Adverse events			X	X	X	X	X	X	X

#### Attendance

Attendance and time spent at each session are recorded by having participants sign in and out of each session, including the precise time at which they enter and leave. The interventionist verifies the times recorded.

#### Satisfaction with intervention

This is assessed with the 8-item Client Satisfaction Questionnaire-8, a briefer version of the larger Client Satisfaction Questionnaire [49]. This measure is frequently used to assess participants’ satisfaction with, and impression of the effectiveness of, the services they received. Items are rated on a four-point scale. This measure has good reliability (alpha coefficient = .93). It is administered at the post-treatment assessment only.

#### Pain

Pain is assessed with the Brief Pain Inventory-Short Form, a 9-item self-report measure that assesses worst, least, and average levels of pain and the degree which pain interferes in activities, mood, relationships, sleep, and enjoyment of life. This measure is widely used with cancer patients, has evidence of reliability and validity, and is considered the preferred method of assessing pain endpoints [50, 51].

#### Fatigue

The Brief Fatigue Inventory assesses self-reported levels of current, worst, and usual fatigue, and interference due to fatigue. The measure consists of 9 items on which respondents choose a number from 0 to 10 to rate each item on the scale. Factor analysis has shown that 75% of the variance on the Brief Fatigue Inventory can be explained by a single factor suggesting that it measures a single construct. This measure has also shown good evidence of concurrent and discriminant validity as well as excellent internal consistency (alpha = .96.) [52].

#### Sleep quality

Sleep quality is assessed by the Pittsburgh Sleep Quality Index, a 19-item self-report measure that produces a total sleep quality score and seven sleep component scores [53]. Higher scores indicate poorer sleep quality. The scale has been widely used with breast cancer patients with internal consistency for the total scale reported as .78 [54].

#### Psychological distress

The Hospital Anxiety and Depression Scale (HADS) is used to assess depression and anxiety symptoms. The HADS is a 14-item, 2-domain (depression and anxiety) scale with evidence of reliability, validity, and responsiveness among cancer patients [55, 56]. Domain scores ≥ 8 indicate either likely depression or anxiety.

#### Mindfulness

The Five Facet Mindfulness Questionnaire-Short Form (FFMQ-SF) assesses different aspects of mindfulness [57]. The FFMQ-SF is a 24-item short form of the Five Facet Mindfulness Questionnaire, a comprehensive measure for assessing mindfulness [58]. The FFMQ-SF has demonstrated reliability and validity as well as sensitivity to change in samples of adults with anxiety and depression as well as fibromyalgia [57].

#### Functional capacity

Patients’ functional capacity is assessed by the 6-min Walk Test administered in a measured corridor according to American Thoracic Society (ATS) guidelines [59]. Patients are instructed to walk at their fastest pace and to cover the longest possible distance over 6 min. This test has been shown to be a reliable and sensitive measure of functional capacity in deconditioned clinical populations and a sensitive test to assess therapeutic response. The test has been successfully used in several prior studies of cancer patients, including patients with advanced disease, with no adverse events [60]. This test has been shown to be a strong predictor of all-cause mortality in lung cancer patients [61].

#### Daily pain, fatigue, positive mood, negative mood, pain catastrophizing, and pain acceptance

Daily variation in pain, fatigue, positive mood, and negative mood is each assessed with a single 0-9 scale item. Similar numeric scales are extensively used in clinical and research settings to measure subjective phenomena and have been found to be valid and reliable [62]. Pain catastrophizing is measured using a well-validated two item measure rated on a 0–6 scale [63]. Pain acceptance is measured using two items from the Chronic Pain Acceptance Questionnaire (CPAQ), the only well-validated measure of pain acceptance [64]. The two CPAQ items were chosen based on their high loadings on each of the CPAQ’s two subscales, willingness to experience pain and engaging in activities despite pain [65].

#### Treatment expectations

We will examine treatment expectations using the 9-item Outcome Expectations for Exercise Scale, in which participants are asked to rate their agreement with potential benefits of physical activity measured on a 5-point Likert-type scale, with 1 indicating no expectations and 5 the highest expectations for exercise. Validity and reliability has been previously established for this measure [66].

#### Safety

Study participants are monitored weekly during the intervention period for the occurrence of adverse events, and at each assessment (baseline, post-treatment, 3 months follow-up, and 6 months follow-up). An adverse event is defined as any unfavorable and unintended diagnosis, symptom, sign (including an abnormal laboratory finding), syndrome or disease which either occurs during the study, having been absent at baseline, or if present at baseline, appears to worsen. Adverse events are recorded regardless of their relationship to the study intervention. Solicited adverse events include psychological distress (HADS score ≥ 15), and heart rate, blood pressure, and oxygen saturation collected during the 6-min Walk Test. In addition, information about muscle soreness is collected at each session of the yoga intervention.

### Statistical analysis

The primary outcome related to feasibility is attendance, which is measured weekly at each intervention session. Adequate feasibility will be indicated by at least 70% of patients attending ≥ 4 of 8 sessions. The primary outcome related to acceptability is quantified by the Client Satisfaction Questionnaire-8 as assessed at the post-intervention evaluation. Adequate acceptability of the yoga intervention will be indicated by ≥ 80% of participants reporting a mean satisfaction score ≥ 3. Secondary outcomes are measurements of change at the post-treatment, 3-month follow-up, and 6-month follow-up evaluations in pain, fatigue, sleep quality, psychological distress, mindfulness and functional capacity. These will be analyzed both as individual time points and in longitudinal analyses. Treatment expectations—including potential pre-randomization selection biases regarding the yoga intervention—will be controlled in analyses with the data from the measure assessing outcomes expectations [66]. We will apply the intent-to-treat assumption for all analyses.

We will also explore within-person associations between yoga practice, outcomes (pain, fatigue, mood) and mediators (pain catastrophizing, pain acceptance) among participants in the yoga condition. Multilevel random effects models will be used to evaluate these associations, with individuals’ mean levels of yoga practice as a control variable, and practice rates person-centered to control for potentially spurious within-person associations [67]. Multilevel modeling is an advanced methodology for integrating data from multiple levels of sampling, such as this study’s two levels (within-persons and between-persons). Multilevel models are particularly advantageous in analyzing data sets with many repeated measures, such as daily diary records [68, 69]. By preserving the rich detail in each individual’s full data set, multilevel models allow for a sensitive independent determination of day-to-day interrelated happenings for each patient, as well as aggregation of individual estimates for reliable results for the average patient. Multilevel models also enable strict control for potential confounds, such as serial autocorrelation in measurements.

## Discussion

In this report we have summarized the challenges of designing a randomized controlled trial of a yoga intervention for metastatic breast cancer patients with long-term follow-up. The challenges we faced in this design included ensuring adequate recruitment including of minorities, limiting and controlling for selection bias, tailoring of the yoga intervention, and maximizing adherence and retention. This project will provide important information regarding the feasibility and acceptability of yoga as an intervention for women with advanced cancer, and valuable preliminary data on the psychological and functional effects of yoga for MBC patients, including the first systematic evaluation of the psychological mechanisms by which a yoga intervention may produce pain relief. This investigation will also establish rigorous methods for future research for testing the mechanisms by which yoga may affect pain, fatigue, emotional distress, and functional capacity in this population.

## Abbreviations

CONSORT: Consolidation of Standards for Reporting Trials
CPAQ: Chronic Pain Acceptance Questionnaire
ECOG: Eastern Cooperative Oncology Group
FFMQ-SF: Five Facet Mindfulness Questionnaire-Short Form
HADS: Hospital Anxiety and Depression Scale
IRB: Institutional Review Board
KPS: Karnofsky Performance Status
MBC: Metastatic breast cancer
RCT: Randomized controlled trial
